# Neoantigen Targeting—Dawn of a New Era in Cancer Immunotherapy?

**DOI:** 10.3389/fimmu.2017.01848

**Published:** 2017-12-19

**Authors:** Thomas C. Wirth, Florian Kühnel

**Affiliations:** ^1^Clinic for Gastroenterology, Hepatology and Endocrinology, Medical School Hannover, Hannover, Germany

**Keywords:** neoantigens, vaccination, personalized cancer immunotherapy, adoptive transfer, mutations

## Abstract

During their development and progression tumors acquire numerous mutations that, when translated into proteins give rise to neoantigens that can be recognized by T cells. Initially, neoantigens were not recognized as preferred targets for cancer immunotherapy due to their enormous diversity and the therefore limited options to develop “one fits all” pharmacologic solutions. In recent years, the experience obtained in clinical trials demonstrating a predictive role of neoantigens in checkpoint inhibition has changed our view on the clinical potential of neoantigens in cancer immunotherapy. Technological advances such as sequencing of whole cancer genomes, the development of reliable algorithms for epitope prediction, and an increasing number of immunotherapeutic options now facilitate the development of personalized tumor therapies directly targeting a patient’s neoantigenic burden. Preclinical studies in mice that support the excellent therapeutic potential of neoantigen-directed immunotherapies have provided blueprints on how this methodology can be translated into clinical applications in humans. Consistently, very recent clinical studies on personalized vaccinations targeting *in silico* predicted neoepitopes shed a first light on the therapeutic potential of personalized, neoantigen-directed immunotherapies. In our review, we discuss the various subtypes of tumor antigens with a focus on neoantigens and their potential in cancer immunotherapy. We will describe the current methods and techniques of detection as well as the structural requirements for neoantigens that are needed for their recognition by T cells and for tumor destruction. To assess the clinical potential of neoantigens, we will discuss their occurrence and functional relevance in spontaneous and hereditary cancers and their prognostic and predictive value. We will present in detail the existing immunotherapeutic options that exploit the neoantigen burden of tumors encompassing both preclinical efforts that provided convincing technological proof-of-concept and the current clinical studies confirming the potential of neoantigen-directed immunotherapies.

## Introduction

Designing tumor therapies which effectively destroy tumors but spare healthy tissues is considered the Holy Grail in clinical oncology. Conventional chemotherapies target tumors but also dividing cells in healthy organs and are therefore frequently associated with significant toxicity. Promising antitumor activity without detrimental side effects, the advent of targeted therapies as a novel class of more tumor-selective oncology drugs initially raised a lot of enthusiasm. Indeed, such targeted therapies led to remarkable remissions in hematologic malignancies as observed with the introduction of imatinib for the treatment of chronic myeloid leukemia (1). In most solid tumors, however, targeted therapies have yielded only limited benefit for cancer patients. Despite the improvement of progression-free survival of cancer patients undergoing palliative treatments, the ultimate goal of significantly improved overall survival could not be achieved.

For more than a century, immunotherapy has been postulated at times as a promising alternative to conventional cancer therapy although clinical proof of its therapeutic efficacy in large patient cohorts was lacking. The perception of immunotherapy as an alternative therapeutic means was mainly driven by case reports of immune-mediated tumor control in cancer patients. Upon occasional observations of tumor regressions in patients in the context of erysipela and high fever, it was William Coley who in late nineteenth century inoculated sarcomas with bacteria (2). Since this was probably the first documented attempt to engage the patient’s immune system in the fight against cancer, William Coley has been referred to as the “father of cancer immunotherapy” (3). Although he reported remarkable outcomes in individual patients, his results were frequently questioned and his methods were later abandoned in favor of the upcoming and “more modern” chemo- and radiotherapy that promised convenient handling and better reliability.

At the beginning of the twentieth century, Paul Ehrlich came up with the suggestion that the immune system is involved in carcinogenesis and in the control of tumor growth during progression (4). Some decades later, these concepts were further corroborated by mechanistic studies in mice. Several groups found that after surgical removal of methylcholanthrene-induced tumors, mice were immune against a second challenge with the same tumor material further supporting the idea of the existence of antitumor immunity (5–7). The discovery of dendritic cells as the relevant cell population for the expansion of T cells in mixed leukocyte reactions (8) and the characterization of the major histocompatibility complexes (9, 10) laid the foundation for a better understanding of the mechanisms of antigen presentation and the mechanisms that govern the induction of cancer-specific cellular immune responses. Supported by methodological advances regarding *in vitro* cultivation of antitumoral cytotoxic T lymphocytes, T cells were suggested as the major effector cell population that specifically responds to tumor antigens in humans (11, 12). Correspondingly, it had been recognized in several clinical studies that the abundance of tumor-infiltrating lymphocytes (TILs) correlates with improved survival of cancer patients (13, 14) indicating that the cytotoxic activity of lymphocytes indeed interferes with tumor growth. The antitumoral potential of T-lymphocytes in patients was later confirmed in a more direct manner. After isolation of TILs, readministration into patients in combination with IL-2 resulted in objective responses in metastatic melanoma (15).

More recently, a number of mechanistic studies in mice have confirmed that the immune system recognizes and attacks tumor cells at all stages of carcinogenesis in a process referred to as immune surveillance. Even premalignant senescent cells are detected and cleared by a process that involves both macrophages and CD4 cells (16). The role of T cells in recognition of tumor cells and control of tumor growth was convincingly shown by Shankaran et al. (17). By comparing the immunogenicity of carcinogen-induced tumors in wild-type and immunodeficient mice, the authors demonstrated that T cell reactivity is the critical determinant of the immunogenicity of mature tumors. How T cells shape the antigenic profile of a tumor in a process referred to as immunoediting was later described in detail in two studies by the groups of Schreiber and Jacks (18, 19). The fundamental influence of the immune system on cancer progression at all stages of cancer development and progression has been acknowledged and consequently designated a hallmark of cancer (20).

However, despite the extensive knowledge of the mechanisms involved in immune-mediated tumor control, successful translation of immunotherapies into the clinic lagged significantly behind these scientific advances. Targeted immunotherapies using peptide- or cell-based vaccines were astonishingly ineffective in clinical trials. Even when the first DC-vaccine targeting prostate cancer (Sipuleucel-T) provided evidence of clinical efficacy the gain in median survival was, similar to the advances achieved with targeted therapies, rather modest without evidence for long-term progression-free survival (21).

Surprisingly, it was a generic approach of T cell stimulation that finally succeeded and initiated the recent success story of cancer immunotherapy. Instead of eliciting a target-antigen-directed immune response in the context of a cancer vaccine, the pharmacologic interference with inhibitory immune checkpoints such as CTLA-4 or the PD-1/PD-L1 axis restored cytotoxicity of preexisting, exhausted cancer-specific T cells. It has to be pointed out that these therapies for the first time in clinical oncology resulted in long-term remissions in advanced cancers (22, 23) that are regarded as complete cures, so far. However, this excellent outcome is limited to a relatively small number of patients, a striking reminiscence of what William Coley observed more than 120 years ago. While the scientific proof of the exceptional therapeutic efficacy of immunotherapy in cancer treatment has been overwhelming, it is also becoming increasingly evident that these immunotherapies are not the long sought “magical bullet” applicable to all cancers. In some tumor entities such as melanoma and Hodgkin lymphoma, over all response rates to either single or combined PD-1/CTLA-4 checkpoint inhibition are encouragingly high (24–26). However, other important cancer entities such as liver cancer and pancreatic cancer are much more resistant to this therapy. Despite all progress, the majority of patients will not experience complete responses, at least when treated with the present options of immunotherapy. Owing to these limitations of current immunotherapies, there is a lot of space for novel therapies that specifically target tumor antigens with well-defined molecular characteristics, thus fulfilling the promise of an individualized immunotherapeutic approach.

## Classification of Tumor Antigens

The initial discovery of the interaction between tumor and immune cells was followed by intensive research to identify the target antigens that were recognized by the adaptive immune system. In 1989, a cell surface glycoprotein of the mucin family, MUC1, which is expressed in tumors in an aberrantly glycosylated form, was described as a tumor antigen that can be recognized by cytotoxic T cells (27). The melanoma antigen family A1 (MAGE-A1) was found to be expressed not only in melanoma but also in other tumor entities whereas it could not be detected in normal tissue except the testis (28). Tumor-specificity of MAGE-A1 is due to the fact that germ line cells lack MHC class I molecules for presentation of the corresponding peptides on their cell surface. MAGE-A1 was therefore a prototypic example of tumor antigens termed cancer testis antigens (CTAs). Another class of tumor antigens are tumor-associated antigens (TAAs) derived from proteins that are overexpressed in cancer but also occur in normal cells. These proteins are frequently involved in transformation-related mechanisms as exemplified by the human epidermal growth factor receptor 2 (Her2/neu) and have also been using as immunotherapeutic targets (29). Since TAAs are also expressed by normal cells, their role as a target antigen for tumor therapy is solely based on their preferential expression in cancers. Their basal expression in normal tissues subjects these antigens to central and peripheral tolerance mechanisms, leading to selection of low-avidity T cells. However, TAA-directed immunotherapy using T cell receptor (TCR)-transgenic, high-avidity T cells may cause severe autoimmunity (30). Compared with TAAs, CTAs such as MAGE-A1 or New York esophageal squamous cell carcinoma-1 (NY-ESO-1) have attracted more attention due to their broad abundance in several tumor entities and their restriction to tumor tissue. Expression CTAs has been shown to be associated with intratumoral lymphocyte infiltration and improved prognosis of cancer patients though these lymphocytes might be functionally impaired (31, 32). It has also been established that adoptive transfer of lymphocytes genetically engineered with an NY-ESO-1-directed TCR is able to induce tumor regression (33).

A highly promising class of tumor antigens are tumor-specific antigens (TSAs). These proteins are not encoded in the normal genome and encompass antigens derived from viral oncogenic proteins (e.g., SV40 from Epstein Barr Virus, or E6/E7 from human papilloma virus) or from proteins that are the result of somatic mutations or gene rearrangements. Whereas the presence of virus-derived proteins is mostly limited to tumors originating from a viral infection process, tumors in general acquire mutations during carcinogenesis and progression, resulting in altered proteins that may serve as neoantigens (34). Neoantigens may be either directly linked to the transformation process (driver mutations) or may occur as a byproduct of increasing genetic instability (passenger mutations) (35). Interestingly, mathematic modeling of the accumulation of mutations during tumor progression suggests that the number of driver mutations may correlate with the total number of mutations in the tumor (36). Neoantigens are probably the most interesting targets for immunotherapies since neoepitopes are not subject to thymic selection and central tolerance. Therefore, the existence of high-avidity T cells is very likely. Furthermore, it has been shown that failure of intrathymic gene expression can give rise to immunogenicity comparable with neoantigens as demonstrated for the melanoma antigen MART-1 (37).

Depending on the position of the mutated amino acid in the sequence of the MHC-bound peptide the non-synonymous mutations differentially impact the quality of the neoantigen. While mutations in anchor positions primarily affect peptide affinity, mutations outside the anchor positions preferentially influence the interaction of the peptide/MHC complex with the TCR. As a consequence, mutations in anchor positions potentially create high affinity epitopes while mutations in the TCR-interacting positions may lead to the recognition of naive T cells which specifically recognize the mutated neoepitope.

First evidence that neoepitopes resulting from non-synonymous mutations are recognized by the immune system as “altered self” was provided by Wölfel et al. The authors identified a p16INK4a-insensitive cyclin-dependent kinase 4 (CDK4)-R24C mutation in melanoma patients as a neoantigen that was a target of CTL responses (38). This mutation of CDK4 disrupts the cell-cycle regulation exerted by the tumor suppressor p16INK4a and is therefore closely associated with carcinogenesis. Consistent with the assumption that neoantigen-directed T cell responses may play a significant role in tumor growth control, it was subsequently demonstrated by the Wölfel group that the antitumor response of autologous T cells in a melanoma patient was predominantly driven by T cells recognizing mutated neoantigens (39). Additionally, neoantigen-directed T cells could be detected in *ex vivo* expanded TILs that had been adoptively transferred in melanoma patients who subsequently experienced a complete tumor regression (40). Together, these findings shed a first light on the use of neoantigen-directed immunotherapies and their clinical potential.

## Identification of Potential Neoantigen Targets for Immunotherapy

According to the results of high throughput cancer genome sequencing it has been firmly established by now that all tumors contain a significant number of somatic mutations (34). However, since neoantigens are the result of sporadic mutations caused by DNA damaging agents and/or random errors of the DNA repair machinery, the set of neoantigens of a tumor is believed to be highly individual (“private”). This feature discriminates neoantigens from tissue-specific, tumor-associated, or other tumor-selective antigens which are considered shared “public” antigens due to their expression in specific organs, their overexpression in cancer or their selective expression in defined tumor entities, respectively. As an exception from this rule, some cancers with high mutational load including microsatellite-instable tumors have been shown to possess a set of shared neoantigens owing to the preferential mutation of distinct genetic regions termed microsatellites (41).

As a consequence of the mostly private nature of neoantigens, potential neoantigen targets can only be identified after analysis of a tumor mutanome by means of whole exome and/or next generation RNA sequencing (42, 43). Depending on the tumor entity and the underlying cause of cancer development these analyses have revealed a wide range for the number of neoantigens detected, ranging from only few mutations in some forms of astrocytoma to several thousands in some melanomas and lung cancers (44).

Of the vast number of non-synonymous mutations detected in tumors only a tiny fraction may be suited for tumor treatment. To enhance the chances for successful immunotherapy a number of critical features of neoantigens have been described that impact on their quality as immunotherapeutic target.

### Expression of Neoantigens in Tumor Cells

As one of the more simple requirements, the target antigen has to be expressed inside the tumor cells. Following transcription from genomic DNA into mRNA, the non-synonymous mutations are translated into the corresponding mutated proteins. Since most studies targeting tumor neoantigens perform next generation mRNA sequencing, mRNA quantity has been used most frequently as a surrogate marker for target gene expression although direct protein measurement is likely to be more accurate. Nevertheless, studies have supported the notion that higher mRNA quantites correlate with both protein quantity and the number of peptide/MHC complexes presented on the tumor cell surface (45). This correlation, however, is rather weak since posttranslational regulation of protein expression and proteasomal processing of the target antigen are neglected.

### Formation of Stable Neoepitope/MHC Complexes

It is known from reductionist antigen models using adoptive T cell transfer that binding affinities between antigenic peptides and MHC class I as well as the binding affinity of the peptide/MHC complex to the corresponding TCR are critical determinants of tumor-directed T cell reactivity and the capability of T cells to reject a tumor (46, 47). It is therefore mandatory to thoroughly assess the ability of neoantigen-derived peptides to form stable peptide/MHC complexes that are able to tightly bind to their cognate TCRs. Following synthesis of the mutated protein, a fraction of the resulting protein is processed by the proteasome, loaded onto MHC class I molecules after transport into the endoplasmatic reticulum by the TAP transporter and presented on the cancer cell surface. The process of proteasomal degradation can be predicted from a number of computational algorithms (e.g., NetChop) but the accuracy of prediction remains to be improved. The expression of neoantigen epitopes with potential clinical relevance has successfully been demonstrated by mass spectrometry (48) but the sensitivity of the method is often limited to epitopes with high expression on MHC. It has been recently shown that the sensitivity of neoepitope detection by mass spectrometry can be significantly increased by monoallelic analysis (49) after retroviral transduction of tumor cells with a specific HLA allele. Due to the limitations of mass spectrometry for less abundant proteins, many studies have not quantitated expression of neoepitopes on tumor MHC complexes but instead focus on *in silico* prediction of neoepitope affinity for a given MHC molecule (50–52). This is justified by the fact that high affinity peptides are more likely to form stable complexes with MHC resulting in increased expression on MHC complexes on the cell surface. Nevertheless, it has to be taken into account that despite high affinity some neoepitopes may never be generated by the proteasome.

To predict the strength of the peptide/MHC binding, the affinity of potential MHC class I epitopes is calculated based on the patient’s MHC haplotype. For this purpose, a number of software programs (e.g., NetMHC and SYFPEITHI) are available that allow accurate prediction of peptide affinity for a neoepitope if the binding properties of the MHC allele are sufficiently characterized (18). Although the reliability of these *in silico* analyses has been questioned (53) the *in silico* binding prediction still represents the first and most important step to identify potential neoantigen targets.

### Activation of Neoantigen-Specific T Cells by Stable Peptide/MHC/TCR Interactions

To generate neoantigen-specific adaptive immune responses the peptide–MHC complexes must be presented on the surface of antigen-presenting cells and interact with neoepitope-specific T cells. In endogenous tumor-specific immune responses, tumor cells undergoing cell death are taken up by dendritic cells which then process endocytosed neoantigens and present the class II neoepitopes on their MHC class II molecules. In parallel, MHC class I neoepitopes are presented on DCs by cross-presentation, a process by which endocytosed proteins after proteasomal cleavage gain access to MHC class I molecules inside the endoplasmatic reticulum (54). This dual requirement for MHC class II molecule presentation and efficient cross-presentation of CD8 epitopes on MHC class I molecules is almost exclusively limited to dendritic cells and among these most prominent to the BATF3-driven lineage (55).

For vaccinations with soluble, short peptides containing MHC class I epitopes (typically 8–10 amino acids in length) or MHC class II epitopes the exogenously administered peptides have to compete with endogenous MHC class I and class II peptides that are already present on antigen-presenting cells. If the neoepitopes are of higher affinity than the endogenous MHC class I, they are able to replace the endogenous peptides directly on the APC surface. For vaccinations in the form of DNA, RNA, or polypeptides/proteins, the target antigen must undergo cross-presentation since these antigen carriers are usually taken up and endocytosed by antigen-presenting cells, thus underlying the same restrictions mentioned above for the endogenous tumor cell-specific T cells responses.

To elicit robust immune responses, the neopeptide–MHC complexes must form an immunological synapse with TCRs on either CD4 or CD8 T cells. Since neoantigens represent “*de novo*” antigens it has been postulated that neoantigen-specific T cells are not subject to central tolerance. As a consequence, neoantigen-specific T cells may not only be of higher functional avidity but may also be more abundant than T cells recognizing autoantigens.

Currently, the methodology for the detection of naive neoantigen-specific T cells in peripheral blood is limited, both in preclinical models and in humans. As an alternative, the number of potentially neoantigen-responsive T cells has been assessed by selective screening of PD-1 expressing, circulating CD8 T cells for their potential to recognize neoantigens (56). However, PD-1 expression on neoantigen-specific T cells indicates beginning exhaustion that could prevent an accurate identification of tumor-specific T cells if intracellular cytokine stainings are performed for T cell detection. Therefore, researchers have advocated the use of tetramers/pentamers to reliably identify neoantigen-specific T cells (57). Using peptide–MHC multimers with DNA barcodes this technology has recently been adapted to allow the large-scale detection of cancer-specific T cells, including T cells specific for neoantigens (58).

However, these methods have a number of caveats to consider. First, detectable neoantigen-specific T cells in patients are likely to be antigen-experienced and more terminally differentiated (59). It is unknown how the quality of these cells compares to truely naive T cells which are endowed with potent replicative capacities. In fact, the number of naive T cells may be reduced in patients with detectable immune responses due to the prior stimulation of the naive T cell pool. Second, systemic immune responses have shown only limited correlation with intratumoral immune responses. Systemic neoantigen-specific T cells may be present due to the lack of trafficking of the neoantigen-specific T cells to the tumor site or because their cognate antigen is not expressed on the tumor cells. Third, neoantigen-specific immune responses detected in the circulation of cancer patients have undoubtedly failed to clear the tumor and their cognate antigen might therefore not represent a favorable target antigen. More studies are needed to assess whether this failure to reject a tumor is primarily due to tumor-derived immunosuppressive mechanisms which could be restored with checkpoint inhibitors or whether this is a T cell intrinsic failure.

Once the neopeptide is sufficiently expressed on MHC and the MHC/peptide/TCR synapse is formed (providing the so-called signal I) the robustness of the ensuing immune response is dependent on additional costimulation (signal II) and secretion of immunostimulatory cytokines such as IFNα and IL-12 (signal III) (60). Since these signals are provided primarily by dendritic cells, efficient T cell priming usually requires signaling through costimulatory molecules and toll-like receptors to induce optimal DC maturation. How this is best achieved in tumor vaccinations remains a matter of debate and much effort is currently devoted to developing strategies that selectively target and activate dendritic cells *in vivo*.

The final step of the neoantigen-directed therapy requires the trafficking of the activated T cell into the tumor tissue and the recognition of the peptide–MHC complex on the surface of the cancer cell by the TCR (61). The T cell must interact with the peptide/MHC complex on the cancer cell and the net result of the TCR/peptide–MHC complex interaction and the activation state of the T cell must result in the production of cytolytic granules. The exact requirements for efficient tumor cell killing currently remain elusive but the affinity of the peptide again seems to play a major role. Interestingly, visualization of the interaction of T cells and tumor cells suggest that the process of tumor cell killing *in vivo* may take much longer than the same process *in vitro*, possibly requiring multiple consecutive hits from cytotoxic T cells (62).

## Role and Frequencies of Neoantigens in Spontaneous Cancers

For the most part neoantigens have been considered random, spontaneous mutations with little overlap between individual patients. Of a wide spectrum of tumors analyzed for their total mutational burden, only few have demonstrated a mutation frequency above 10/megabase DNA. In these tumors, the few neoantigens are randomly distributed throughout the genome which has led to a view of neoantigens as entirely “private” antigens. At second sight, however, different classes of mutagens have been shown to induce non-random changes in genomic DNA sequences. As an example, UV light induces C to T transitions in dipyrimidine contexts whereas tobacco smoke preferably induces G to T transitions. For tumors with low mutational load this bias in DNA alterations is not sufficient to result in recurrent non-synonymous mutations. In tumors with a high mutational load like melanoma, however, a C to T transition in the gene RQCD1 has been shown to result in a recurrent P131L mutation with a prevalence of 4% in a population of 715 melanomas (63). Similarly, large scale whole-exome sequencing in 619 colorectal cancer patients revealed preferential mutations in BCL9L, RBM10, CTCF, and KLF5 (64). Of interest, some of these genes are known driver genes in other tumor entities pointing toward a preferential selection of genetic alterations that promote tumor growth. These results suggest that although most neoantigens in sporadic tumors are indeed “private,” both the type of mutagen and a selection for driver mutations can result in recurrent neoantigens whose frequencies are currently underestimated. These results warrant further large-scale whole exome analyses in other tumor entities to corroborate the findings from melanoma and colorectal cancer patients.

## Role of Neoantigens in Hereditary Cancers with DNA Repair Deficiencies

Cancers with hereditary defects in genes involved in DNA repair are characterized by high frequencies of non-synonymous mutations. As an example, patients with Lynch syndrome harbor mutations in DNA mismatch repair genes resulting in thousands of neoantigens per tumor. In patients with Lynch syndrome, the mismatch repair deficiency does not only induce DNA base exchanges but results in the accumulation of insertions or deletions at mutation-prone DNA hot spots with repetitive base pair sequences [referred to as microsatellite instability (MSI)]. As a consequence, whole exome analyses of tumor samples from patients with Lynch syndrome have revealed a number of recurrent frameshift mutations in genes with microsatellite sequences. Similar to the reported genetic alterations in sporadic tumors some of these frameshift mutations presumably target genes involved in tumor development, particularly genes with tumor-suppressor function including TGFBR2, BAX (65, 66), CRTC1, BCL9, JAK1, and PTCH1 (67). The preferential mutation of genes with microsatellite sequences in patients with Lynch Syndrome has led to the identification of a set of genes with high mutation frequencies in MSI patients (TGFBR2, AIM2, HT001, and TAF1B) which have been used as a vaccine in a clinical trial (68). Although prototypic, colorectal MSI cancers represent only one example of tumors with mismatch repair deficiencies. Highly immunogenic mutations have, for example, been reported for other MSI tumor entities including gastric cancer, ovarian cancer, glioblastoma and others (69), Polymerase ε-mutant glioblastoma (70), colorectal, and endometrial cancers (71, 72), as well as BRCA-mutated ovarian cancer (73). For these tumor entities, recurrent neoantigens that are shared between patients may represent prime targets for immunotherapy, in particular frameshift mutations which typically harbor multiple novel epitopes that are recognized across various MHC haplotypes.

As a potential caveat, the large mutational burden in patients with mismatch repair deficiencies seems to greatly accelerate the formation of immune escape variants. In patients with MSI-tumors, defects in antigen presentation have been detected in MHC molecules and in molecules associated with MHC expression at high frequencies (74). These MSI cancers may exhibit greatly reduced sensitivity to T cell-mediated killing, a potential caveat that has to be considered for the appropriate design and timing of vaccines targeting MSI cancers.

## Prognostic and Predictive Value of Neoantigens

Microsatellite instable tumors are increasingly recognized as a subset of tumors with distinct prognostic and predictive features. In patients with colorectal cancers, MSI tumors are overrepresented in early stage cancers but underrepresented in metastatic disease. This feature of MSI tumors has been attributed to the presence of high numbers of immune cells in MSI tumor specimens which may limit local tumor recurrence and systemic spread. Patients with MSI-H colorectal cancers UICC stage II have been shown to have a favorable prognosis compared to patients with MSI-L or microsatellite stable (MSS) tumors (75, 76), depending on the individual mutation and concomitant allelic losses (77, 78). In metastatic stage IV colon cancer the prognosis of patients with MSI colorectal cancer is similar to patients with MSS tumors but associated with better survival in patients with peritoneal metastases and lower survival in patients with lymphatic or blood-borne metastases (79).

The better prognosis of patients with MSI tumors has a direct impact on the treatment of this patient population after tumor resection. Owing to the lower frequency of local recurrence and systemic spread after resection, adjuvant therapy for UICC stage II MSI colon cancer is not recommended. For UICC stage III patients, the usefulness of adjuvant therapy in MSI patients is still a matter of debate, with some studies arguing in favor of adjuvant therapy (80) and others against it (81).

More recently, the clinical success of checkpoint inhibitors in melanoma patients has revealed an additional predictive role of the mutational load in patients treated with either anti-PD-1 or anti-CTLA-4 antibodies (82–84). These clinical effects have been suggested to be due to neoantigen-specific immune responses which are restored upon administration of checkpoint inhibitors (85, 86). In patients with MSI tumors, both PD-L1 expression on cancers and PD-1 expression on TILs is increased thus providing a molecular basis for the better clinical response to anti-PD-1 therapy (87–89). The predictive role of neoantigens may extend to other immunological treatments including adoptive T cell therapy. Although this has not yet been demonstrated convincingly, neoantigen-specific immune responses have been detected in TILs (90, 91). In these patients, neoantigen-specific immune responses show evidence for robust clonal expansion indicating that the quantity and quality of neoantigens in the expanded T cell pool could influence the therapeutic efficacy of TIL transfer.

## Neoantigen-Directed Tumor Therapies

The notion that neoantigen-specific T cell responses are involved in tumor growth control in patients raised significant interest in identifying specific neoantigens as suitable targets to facilitate the design of tumor-directed vaccines. A particularly attractive kind of neoantigens are those that represent relevant mutation in tumor driver genes. It has been widely assumed that immunotherapies targeting noepitopes originating from oncogenic driver mutations may induce antitumor responses in a most effective manner since they are most likely essential for tumor survival and are homogenously expressed throughout the tumor tissue. Consequently, investigations initially focused on neoepitopes derived from well-known mutations in prominent oncogenes such as KRAS mutated at codon 12, or mutated p53 (92, 93). An oncogenic alteration that frequently occurs in melanoma is the V599E missense mutation in the kinase domain of BRAF giving rise to a mutation-specific epitope that can be recognized by T cells (94, 95). Furthermore, in particular hematological malignancies mutations in either JAK2 (JAK2V617F) or mutations in exon 9 of calreticulin are abundant incidents giving rise to spontanoues T cell responses (96, 97). These mutations could be interesting targets for immunotherapy as well as recently described amino acid exchange in the histone H3 gene (K27M) that is frequent in glioma (98, 99). A peptide vaccine against this mutation was capable to effectively induce mutations-specific immune-responses in a MHC-humanized mouse model (100). Also in humanized mice, it has been demonstrated that a vaccine targeting mutant isocitrat dehydrogenase-1 (IDH1R132H) induced mutation-specific T cells and was able to control the growth of preestablished tumors (101). A corresponding vaccine is currently investigated in clinical trials in glioma patients.

Although the abovementioned mutations represent rather frequent genetic events the abundance of shared neoepitopes is significantly reduced by the huge HLA diversity rather low and inter-individual overlap is limited. The feasibility to develop broadly applicable vaccines has been recently estimated by a genomic analysis and epitope prediction of more than 63,000 tumors across multiple tumor entities and for the most common HLA A/B subtypes (102). Hypothesizing that sets of carefully selected neoantigens could allow for development of broadly applicable vaccines these calculations revealed that neoantigen targets still remain highly diverse even when regarding major and frequent driver mutations. Nevertheless, the fact that shared neoepitopes are not fully private compared to other mutation-derived epitopes is an important technical advantage, and, once established corresponding vaccines could function as a valuable backbone in more complex multiepitope targeting approaches to prevent the rise of escape mutants.

**Figure 1:**
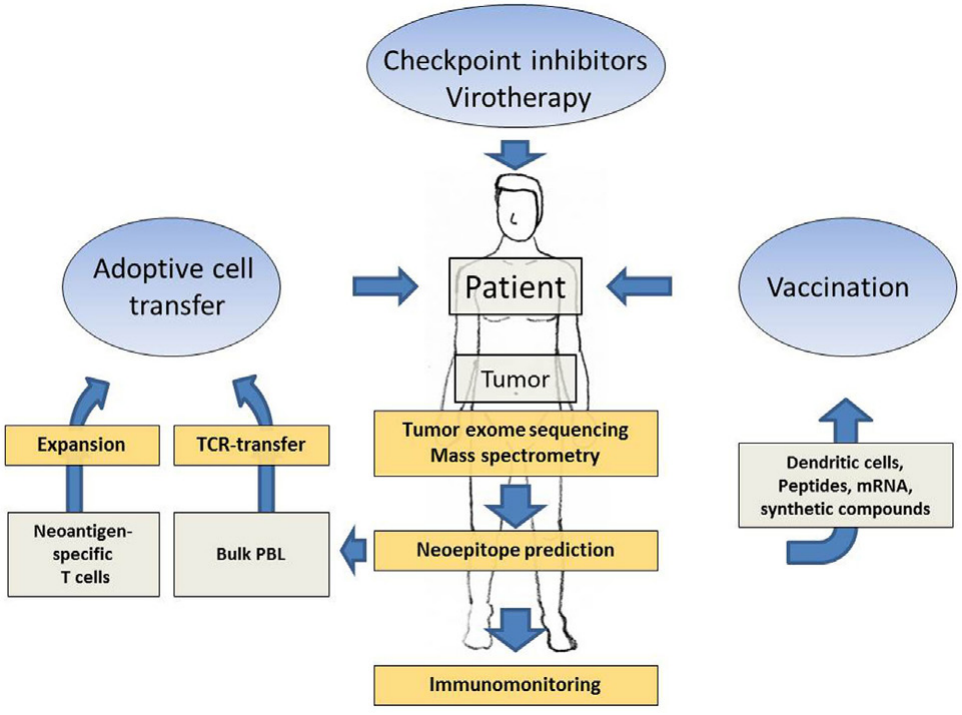
Schematic presentation showing how patient-specific tumor sequencing data can be translated into several options of neoantigen-directed immunotherapies. Next generation sequencing of tumor tissue, neoepitope prediction and mass spectometry analysis, supported by immune activating interventions such as checkpoint inhibition and virotherapy, provide the methodology to delineate promising neoantigenic patterns as targets for tailored immunotherapies. Neoantigen-directed immunotherapies include adoptive cell transfer approaches and tailored vaccines.

Several technological advances in parallel opened up new avenues for the discovery of neoantigens and their potential use as target antigens in cancer immunotherapy. Next generation sequencing facilitated the exploitation of whole tumor exomes and revealed that in all tumors the mutated genome encodes for a variable but significant number of non-synonymous mutations and thus potential neoantigenic epitopes (34). Furthermore, raw DNA sequencing data can be rapidly processed *in silico* and algorithms are available that help to predict neoepitopes. These technologies therefore promise to achieve the identification of suitable neoepitope candidates for patient-specific immunotherapy within acceptable time, being one of the most critical requirement for patients with progressive tumor growth. The correlation of therapeutic efficacy of checkpoint inhibitors and the neoantigenic load clearly demonstrated that neoepitopes could play a prominent role also in more target-selective approaches of cancer immunotherapy. Several preclinical studies developed and simulated workflows including tumor exome mining and neoepitope prediction, eventually followed by methods to confirm truely immunogenic neoepitopes within the predicted pool, aiming at the development of a personalized immunotherapy. Just recently, the first results from clinical studies which applied these preclinically established methods to real-life therapeutic settings in humans have been reported. The promising clinical results will be described in more detail in the following subchapters. Furthermore, the full spectrum of immunotherapies targeting neoantigens in cancer patients is summarized in Figure 1.

### Checkpoint Inhibitors As a Systemic Approach to Activate Neoantigen-Directed T Cell Responses

Antagonizing coinhibitory molecules has shown great success in treatment of some cancer entities even at advanced stage (22, 23). Checkpoint inhibitors are generic stimulators of T cell responses and part of their activity is therefore directed against neoantigens that can be detected by T cells. A potential involvement of neoepitopes in therapeutic efficacy in melanoma has early been assumed since the mutational load in this particular tumor entity is rather high. To prove the relevance of neoantigens as immunotherapeutic targets the contribution of neoantigens to the observed therapeutic responses following application of checkpoint inhibitors has been assessed by a number of studies. First evidence that there is indeed a positive correlation between the mutational burden in tumors and the observed response came from checkpoint inhibitor studies using either PD-1/PD-L1 or CTLA-4 inhibitors. After tumor exome sequencing and data processing with NetChop and NetMHC algorithms, van Rooij et al. showed the expansion of a T cell response directed against a mutated version of ATR (ataxia teleangiectasia and rad3 related) in a melanoma patient after therapeutically effective ipilimumab treatment (85). Snyder et al. directly investigated the correlation of the mutational load in melanoma and therapeutic response to CTLA-4 inhibitors ipilimumab and tremelimumab (82). They found that the mutational load was indeed associated with the degree of clinical benefit. More detailed investigations by genome-wide neoepitope analysis and patient-specific HLA-typing allowed the description of specific neoantigenic “landscapes” that are present in tumors responding to this therapy. In parallel, Rizvi et al. correlated the clinical benefit of the PD-1 inhibitor pembrolizumab with the mutational load in a patient cohort with non-small cell cancer patients with a wide range of neoantigen frequencies due to cigarette smoking. They found that the non-synonymous mutational load in tumors was associated with improved objective response, durable clinical benefit and progression-free survival (83). Therapeutic efficacy correlated with several parameters resulting from the mutational load such as a mutation-rich molecular smoking signature, higher neoantigenic burden, and DNA repair pathway mutations. Consistent results were described by a phase 2 study in colorectal cancer patients with mismatch repair deficiency which harbor hundreds to thousands of mutations (103). Tumors with mismatch repair deficiency showed a significantly higher progression-free survival after pembrolizumab therapy compared to mismatch-proficient tumors. These observations confirmed the role of mutational burden as a predictive marker in checkpoint therapy of MSI patients and suggest an important role for neoantigen-directed immune reponses in patients with highly mutated tumors. Nevertheless, checkpoint inhibitor studies have also demonstrated that accurate prediction of immunoresponsiveness remains challenging since a significant number of patients failed to respond to checkpoint inhibition despite a high mutational load. Future studies are therefore required to reliably discriminate predictive from non-predictive mutations in patients undergoing checkpoint inhibition and to convincingly demonstrate the role of neoantigen-directed adaptive immune responses.

### Preclinical Studies Targeting Neoantigens

Several studies in mouse models delivered blueprints how neoantigen-directed immunotherapies can be applied in the future for effective immunotherapy of cancer. Through investigations in mouse models it was demonstrated that neoepitopes are important targets of the immunoediting processes during carcinogenesis (18, 19) which decisively shape the immunogenic profile of a mature tumor. The Schreiber group identified and validated neoepitopes in highly immunogenic carcinogen-induced sarcomas including neoantigens that enabled tumor rejection such as spectrin-β R913L, or were involved in tumor rejection in response to checkpoint inhibition such as neoepitopes derived from mutated LAMA4 or mutated ALG8 (104). In parallel, investigations were undertaken to find out how these neoantigen-derived immunogenic profiles can be determined and translated into suitable targeted immunotherapies. The Sahin group used next generation sequencing to identify 962 non-synonymous point mutations in B16F10 melanoma cells from which 563 were found in expressed genes (42). Next, the researchers investigated the actual immunogenicity of 50 selected peptides harboring most promising candidate mutations according to prediction with NetMHC. One third of these peptides were indeed immunogenic with 60% preferential activity against the mutated peptide compared with the wild-type equivalent. In transplant tumor models, vaccinations with these peptides conferred antitumor activity in protective as well as therapeutic settings. Together with the findings by the Schreiber group described above these observations clearly demonstrated the feasibility of bioinformatic evaluations of entire neoepitope spectra that can be used as raw material to identify therapeutically relevant immune responses targeting neoepitopes. In a different approach, Yadav et al. further optimized the accuracy of immunogenic neoantigenic peptide identification by including mass spectrometry of peptides present on MHC class I molecules (105). For their purpose, the authors investigated two widely used tumor models including the murine colon carcinoma cell line MC38. Using whole-exome and transcriptomic sequencing as well as MHC binding prediction, they found 1,300 amino acid changes of which 13% were potential MHC class I binders. A small fraction of these candidates were indeed confirmed by mass spectrometry. The circle of candidates was further narrowed down by molecular modeling of the peptides bound in the groove of MHC class I. Only those peptides that exposed the mutation to the exterior were considered immunogenic. Those included the strong H-2D_b_ epitopes of mutated Reps1 and Adpgk, and the H-2K_b_ epitope of Dpagt1. Remarkably, vaccinations with peptides predicted by this combined *in silico* prediction/mass spectrometry approach yielded therapeutically active T cell responses thus impressively confirming its excellent accuracy. Central aims of these strategies were not only to show the feasibility of *in silico* prediction of neoepitopes within acceptable time but also to demonstrate the accuracy and reliability of the chosen *in silico* approach in identifying immunogenic neoepitopes. Mass spectrometry was a first powerful analytical step to narrow down the number of therapeutically relevant neoepitodes with an additional validation step. However, the more stringent the selection criteria are *in silico* or *in vivo*, the higher the risk to omit relevant neoepitope candidates for targeted therapies. As an example, the nature of the chosen immunotherapy may impact on the quality of neoepitope responses as Gubin et al. demonstrated in their study when showing treatment-specific transcriptional alterations in neoepitope-specific CD8 T cells after CTLA-4, or PD-1 checkpoint inhibition, respectively (104). As an alternative means to identify neoepitope-specific CD8 T cell with potential relevance in immunotherapeutic treatments, we have pursued an alternative strategy including neoepitope prediction and confirmation of immunogenicity using intratumoral application of oncolytic viruses. To this end, we analyzed the spectrum of candidates for neoepitope-specific CD8 T cell responses in murine CMT64 lung cancer cells that are highly resistant to immunotherapy such as systemic PD-1 blockade (106). Similar to the aforementioned studies, next generation sequencing and data processing facilitated the detection of 274 non-synonymus mutations. The corresponding peptide sequences were analyzed by the SYFPEITHI algorithm for CD8 T cell epitope prediction to yield 44 neoepitope candidates that potentially bind to MHC class I with high affinity. Among those, five neoepitope-specific responses directed against the mutations H2Q2-D244E, Ndufs1-V491A, Rab13-K196N, Ppat-I208M, and Gsta2-Y9H were identified in peripheral blood following intratumoral application of an oncolytic adenovirus in all investigated individuals. Interestingly, when intratumoral virotherapy was administered together with systemic PD-1 checkpoint inhibition, a strong broadening of the neoepitope spectrum with improved antitumor efficacy was observed including neoepitope-specific responses that were neither detectable after PD-1 blockade nor after virotherapy, when applied as monotherapies. The use of tumor selectively replicating viruses is therefore not only an effective means to lyse tumors. Our observations also demonstrate that viral oncolysis mimics the effect of a vaccine that covers the complete antigenic spectrum of the target tumor, including neoantigens. Consequently, application of oncolytic virotherapy may not only be used as direct tumor therapy but may also serve as a method to validate the responsiveness of tumor-specific T cell clones to a predicted neoepitope, either for tracking and assessing the success of therapy or for facilitating the design of additional immunotherapeutic means to further enhance responding neoepitope-specific T cell responses. Yet another alternative prediction method considered the difference in NetMHC score between a neoepitope and the unmutated counterpart together with the overall affinity of the peptide bound to MHC class I. By applying this method, Duan et al. were able to detect unique neoepitopes that provided substantial tumor protection (107). Interestingly, the authors also found neoepitopes with rather weak affinities that were lower than the affinity threshold that is usually considered sufficient for effective interaction. Though mechanistic studies have suggested that high affinity neoepitopes are mandatory for tumor rejection, it will need further investigations to discriminate how several weak or moderate avidity CD8 T cell responses may cooperate in tumor rejection. Although responses against a single, low-affinity neoepitope might be insufficient for tumor rejection, multipronged responses may develop enough cumulative antitumor efficacy required for rejection and at the same time prevent the generation of escape variants.

A further critical aspect is the contribution of neoepitope-specific immune responses by CD4 T cells. The contribution of CD4 T cells to antitumor effects is known from depletion studies and the presence of antitumoral antibodies (104, 108). The observed control of tumor growth in experimental tumor models harboring transposons for CD4 and CD8 neoepitopes also suggest a mechanistical role of CD4 and CD8 T cell interaction in cancer immunosurveillance (109). The relevance of CD4 T cell neoepitopes has been shown in humanized mouse models and in patients. Schumacher et al. demonstrated that after peptide vaccination of mice transgenic for human MHC class I and II with a mutated peptide of isocitrate dehydrogenase 1 (IDH1), mice developed effective MHC class II-restricted, mutation-specific antitumor immune responses resulting in growth control of tumors expressing mutated IDH1 in a CD4 T cell-dependent manner (101). The Rosenberg group confirmed the therapeutic relevance of a CD4 T cell neoepitope in a patient with metastatic cholangiocarcinoma following adoptive cell transfer (110). Linnemann et al. investigated and validated neoantigen-specific CD4 T cell responses in melanoma patients and found that these responses are indeed present but rather rare events (90). In a study in mice, the Sahin group found surprisingly that vaccinations using long peptides containing predicted CD8 T cell neoepitopes resulted in effective, tumor-directed T cell responses that were vastly dominated by neoepitope-specific CD4 T cell responses (111). These findings might be due to the method used for prediction or to species-specific effects. Also, it has to be taken into consideration that prediction of MHC class II epitopes is in general more error-prone than prediction of MHC class I epitopes. This is due to the fact that peptides are more loosely bound to the groove of MHC class II and a more variable size is tolerated making *in silico* prediction more demanding compared with prediction of CD8 T cell epitopes.

Whereas multiple studies support the usefulness of neoepitope prediction for the design of immunotherapies the limitations of this method have been far less defined. A study by Martin et al. suggest that this could be the case in tumors with moderate to low mutational burden. This has been assessed by whole exome and transcriptome sequencing on ID8-G7 cells (112). The authors identified 39 transcribed missense mutations and applied corresponding peptide vaccines in mice. Whereas 7 of 17 neoepitope-specific vaccines, directed against predicted MHC class I binding mutations, induced robust mutation-specific T cell responses, none of the vaccines yielded a therapeutic benefit in tumor-bearing mice illustrating the limits of neoantigen-directed immunotherapy.

A specific future requirement for neoepitope response prediction in immunotherapy should include the reliable coverage and definition of a neoepitope-specific T cell responses capable of tumor rejection. This remains a challenging task when only relying on *in silico* approaches. Certainly, a stringent immunomonitoring is required to detemine neoepitope-specific T cells that actually respond to therapy (113). Much of the preclinical work using *in silico* epitope prediction that has been presented up to now has been performed in inbred animal models with relatively little pathogen exposure reflecting rather unexperienced “naive” individuals. It is therefore a general question in how far the obtained data reflects the situation in humans patients considering the vast diversity of the “immunome” in immunologically experienced cancer patients. Therefore, additional analytical steps are urgently needed that take into consideration how the human immune system is altered in aged and immunologically experienced cancer patients. This should facilitate the design of a tailored therapy that fits the needs of a truly personalized neoantigen vaccine.

### Adoptive Cell Transfer Strategies

Next generation sequencing techniques and neoepitope prediction have also facilitated more precise investigations of the specificities of TILs and the design of neoantigen-directed T cells for adoptive transfer immunotherapies. Adoptive transfer of tumor-directed immune effector cells such as TILs represents a classical approach to target tumor antigens for cancer immunotherapy. A striking advantage compared with active immunization or checkpoint inhibition is that tumor-reactive cells can be identified and then expanded *in vitro* to large numbers before giving them back to the patient in combination with IL-2 (114). As a potential limitation, the method requires an invasive procedure to obtain material for isolation and growth of the desired TILs. Furthermore, TILs may contain exhausted, terminally differentiated populations that limit their use in adoptive T cell therapy approaches or T cells that do not recognize tumor antigens. An alternative is to redirect peripheral blood lymphocytes to tumors by transduction with heterologous TCRs to facilitate tumor recognition. First clinical trials with ACTs using genetically engineered TCRs against MART-1 or NY-ESO1 showed objective tumor responses, but also “off-target” toxicities (33, 115). Since neoantigens are bona fide TSAs, the adoptive transfer of neoantigen-directed T cells promises antitumoral activity without off-target effects and thus reduced adverse events. Correspondingly, various therapeutic approaches have been reported that successfully translate these principles to adoptive transfer of neoepitope-directed T cells. The Blankenstein group (116) generated transgenic T cells expressing a TCR directed against a known immunogenic mutation in CDK4 which results in two mutant isoforms of CDK4. In an MHC class I humanized mouse tumor model, the authors showed effective expansion of T cells and IFN-y expression. Interestingly, the response to these two isoforms was dramatically different indicating the highly variable quality of neoantigens to serve as T cell targets. Using transcriptomic sequencing of a UV-induced tumor, Leisegang et al. identified a mutation in p68, a coactivator of p53. This mutation turned out to be a well suited neoepitope since it reflects a trunk mutation and binds to MHC with high affinity. TCR-transgenic T cells recognizing this neoepitope were capable of eradicating established tumors. However, when the antigen was autochthonously expressed, T cell pressure promoted the emergence of escape variants (117). Immune escape was prevented when expression of the neoantigen was warranted in all tumor cells or when additional immunotherapeutic means such as irradiation were applied. The emergence of escape variants parallels clinical experience with molecular targeted therapies and strongly recommends the development of multi-targeted immunotherapies to prevent immunotherapy failure.

To engage a significant number of functional neoantigen-directed T cell specificities, the Rosenberg group first enriched neoantigen-specific TILs prior to isolation of the corresponding TCRs. The information from tumor exome sequencing and epitope prediction was used to generate tandem minigene constructs harboring the corresponding mutated sequences for expression of the corresponding neoepitope peptides in dendritic cells. Coincubation of these DCs with TILs resulted in enrichment of neoantigen-reactive T cells facilitating the isolation of neoantigen-reactive TCRs for later transduction of peripheral blood lymphocytes (118). The authors thus presented a feasible method to generate functional and effective neoantigen-reactive T cells for future adoptive cell transfer immunotherpies.

This group also proposed the TCR transfer into peripheral blood lymphocytes by electroporation of sleeping-beauty transposons encoding patient-derived TCRs reactive against particular neoantigens. In a murine context, these T cells harboring TCR encoding transposons were able to rapidly expand and to mount polyfunctional responses against the cognate neoantigens suggesting sleeping beauty mediated transposition of mutation-specific TCRs as a suitable method to generate personalized adoptive T cell therapies (119).

A limiting factor of neoantigen-directed immunotherapy appears to be fact that only a minority of predicted neoepitopes is recognized by autologous TILs. To address this bottelneck, Strønen et al. suggested strategies to complement the spectrum of T cell responses in individual patients using the TCR repertoire of healthy donors. In these heterologous T cell repertoires they discovered neoantigen-recognizing T cells responding to predicted neoepitopes in tumor patients that were neglected by the patients autologous T cell repertoire. T cells redirected with the TCRs from donor-derived T cells were then able to effectively recognize the patient-derived melanoma cells (120).

A generally attractive method to redirect T cells to cancer cells is the use of chimeric antigen receptors (CARs). In CARs, the ligand for the molecular target is usually a single chain variable fragment (scFv) derived from a target-binding antibody. The use of CARs circumvents some problems associated with the use of TCR transfer such as mixed chimerism, unwanted off-target specificities and MHC downregulation in target cells. Posey et al. have developed a CAR that recognizes the tumor-specific glycoform of MUC1, a TSA already described in the introduction (121). Anti-MUC1 CAR T cells demonstrated effective cytotoxicity and tumor growth control in xenograft models of leukemia and pancreatic cancer. However, the glycosylated form of MUC1 is present in various cancers and is therefore not fully representative of mutation-derived neoantigens as described in the previous chapters. It remains an open question whether the CAR approach can be reasonably translated into highly personalized immunotherapies targeting mutation-derived neoantigens.

### Neoepitope-Directed Vaccination and Current Clinical Trials

Although the history of clinical success of cancer vaccines has so far been rather disappointing, vaccines remain a promising tools for targeted immunotherapy. It is currently unknown whether vaccines with neoantigens are able to augment pre-existing responses in patients which have failed to reject a tumor. It has been shown by Carreno et al. in melanoma patients that a dendritic cell vaccine directed against a number of predicted neoepitopes indeed led to an increase in naturally occurring neoantigen-specific responses. Most importantly, the vaccination was able to induce epitope spreading by triggering *de novo* neoantigen-specific responses with diverse TCR usage (52). These observations showed the clear benefit of vaccinations with regard to the breadth of the immune response and the clonal diversity of neoantigen-directed immunity.

Two recent clinical studies have provided further proof-of-concept to translate neoepitope prediction into personalized cancer vaccine formulations which induce effective tumor responses in patients with advanced cancer (50, 51). Sahin et al. have administered a highly personalized RNA-based vaccine in 13 patients with advanced melanoma. The personalized vaccines were set up by an RNA reflecting five connected 27mer peptides harboring MHC class I and class II neoepitopes with high binding prediction scores. The researchers showed that vaccination led to rapid expansion of neoepitope-specific responses with central and effector memory phenotypes. Vaccine-dependent T cell infiltration and neo-epitope-specific tumor cell killing was confirmed in resected tumor material. With regard to the clinical outcome, the authors observed a reduction in metastases and an objective response in two out of five patients. One of the two responding patients later relapsed due to the loss of β2-microglobulin indicating an adaptive immune escape of the tumor. Strikingly, the authors found a complete response when vaccination was combined with checkpoint inhibition. In a second study, Ott et al. subcutaneously applied a personalized, peptide-based vaccine with polyIC:LC (Hiltonol) in six patients with advanced melanoma (51). Here, up to 20 neoepitopes were selected, based on previous determination of the neoepitope binding affinity to HLA molecules. After vaccination, the patients developed multifunctional CD4 and CD8 T cell responses. Of these patients, four had no recurrence at month 25 after treatment. The two patients with recurrent disease were additionally treated with the PD-1 blocking antibody pembrolizumab and experienced complete regressions while significant expansion of the neoepitope-specific T cell repertoire was observed. Although these findings need to be further corroborated in larger clinical studies, they prove feasibility and safety of this approach and promise excellent synergy when combined with subsequent checkpoint blockade.

## Resistance Mechanisms

The development and systemic spread of cancer represents a failure of both the innate and the adaptive immune system. Immune cells have been shown to control even the earliest events of malignant transformation by induction of senescence in pre-malignant cells in a CD4-dependent manner (16). Due to the constant interaction of malignant and immune cells the tumor undergoes a process called “immunoediting” consisting of the three distinct phases elimination, editing and escape (17, 122). During the elimination phase, innate and adaptive immune cells recognize malignant cells and eliminate them to prevent the formation of tumors. In case this early elimination fails, tumor cells may enter a state of dormancy in which both systemic spread and complete elimination of the malignant cells is prohibited. Eventually, cancer cells may escape from this equilibrium phase by evading recognition of the immune system, resulting in local formation of cancers, tumor recurrence and eventually systemic spread. In clinically apparent tumors a number of escape mechanisms have been shown that prevent recognition by the immune system.

First, tumors may silence the expression of the recognized antigen. Similar to tumors with only heterogeneous expression of the target antigen, this escape mechanism results in the development of tumor cells devoid of the target antigen. Loss of the target antigen has been convincingly shown in mice and cancer patients undergoing immunotherapy (123, 124). This phenomenon of immune escape is of particular importance in mono- or oligoclonal immune responses and in immunotherapeutic approaches that target bystander mutations. Under these circumstances, cancer cells can easily escape recognition by the T cells without detrimental effects on cancer cell growth by downregulating the expression of target genes if their functions are dispensable for cell viability.

Second, antigen presentation can be negatively affected. Tumors may downregulate the expression of MHC molecules, either by allelic loss or downregulation of protein transcription or translation (125). However, the frequency of MHC-downregulation is difficult to estimate since MHC expression appears to be extremely heterogenous when stained in biopsy material, also when regarding primary tumors and metastases (126, 127). A further reason for reduced antigen presentation could be the loss or reduced expression of genes that are part of the antigen-processing machinery such as the transporter associated with antigen processing (TAP) (128). The impact of these mechanisms of immune evasion has been convincingly shown in cancer patients and may result in the formation of cancer cells that are no longer subject to surveillance by CD4 or CD8 T cells (129). However, these cancer cells can still be targeted by natural killer cells or CAR T cells which are able to recognize cells devoid of MHC molecule expression or can be eliminated indirectly in a processes referred to as bystander killing (130).

As a third escape mechanism tumors may create a local milieu of immunosuppression that prevents the formation of an immunological synapse between cytotoxic T cells and the tumor cells. This may be achieved simply by preventing access of T cells to tumor cells inside the affected organ, for example by extensive proliferation of tumor-associated stromal structures (131). Alternatively, tumors may orchestrate the accumulation of immunosuppressive cell populations (e.g., regulatory T cells and myeloid-derived suppressor cells) into the tumors that negatively affect T cell trafficking, expansion or differentiation of T cells into functional cytotoxic T cells, leading to functional exhaustion and ultimately deletion of cancer-specific T cell clones (132). This resistance mechanism is of particular importance in solid tumors with a strong stromal component which often takes an active part in the suppression of adaptive immune responses (133, 134). Upregulation of PD-1 on T cells has been shown to be one of the phenotypic hallmarks of cancer-induced T cell exhaustion thus laying the foundation for the ground-breaking checkpoint inhibitor studies in patients with solid tumors (135).

## Considerations on Design of Therapeutically Efficient Neoantigen Vaccines

The mechanisms of immune escape described above have to be considered in the design of immunotherapy trials targeting neoantigens. To minimize the chance for the development of escape variants current vaccination trials aim at inducing polyclonal immune responses against multiple epitopes, in some cases up to 20 neoepitopes in a single vaccination (51). However, these epitopes are typically derived from bystander rather than driver mutations due to the limited number of functionally activating somatic mutations in driver genes. The outgrowth of tumor cell clones without MHC surface expression poses a yet unsolved problem to neoantigen-targeting vaccination approaches. One of the most promising strategies to prevent the formation of MHC-negative clones could be to minimize the time for the tumor to adapt to the adaptive immune response by mounting rapid, polyclonal T cell responses (“hit hard and early” strategy). However, this strategy does not take into account that MHC-negative cancer clones could be present even before the vaccination. This has to be considered since MHC downregulation appears to be a rather frequent event in response to tumor immune recognition. If loss of MHC expression represents a stochastic event, reduction of tumor mass before the vaccination or adjuvant vaccinations after tumor resection could represent a possible solution.

## Future Outlook

In the past years, immune responses targeting neoantigens have gained considerable attraction due to a number of clinical reports that have demonstrated the potent clinical effect of adaptive immune responses against these TSAs in cancer patients. As summarized above, the impact of neoantigen targeting extends from a predictive role in checkpoint inhibition to convincing clinical effects in individual patients after adoptive cell transfer and culminates in the recent success of personalized neoantigen vaccines in melanoma patients (50, 51). After a series of disappointing vaccination attempts, these results currently spur the hope that the goal of personalized immunotherapy is finally within reach. However, for a broad application of neoantigen-targeting immunotherapies in humans there are still a significant number of obstacles that have to be addressed and solved in the near future. Cancers treated by personalized immunotherapies in the form of adoptive CTL transfer or vaccinations are exposed to a high selection pressure favoring the evolution of escape variants. In fact, some of the very first reports of neoantigen-directed vaccines have already demonstrated a number of resistance mechanisms of solid tumors. As one example, loss of neoantigens with heterogeneous expression inside the treated tumor has been shown to result in the selection of subclones devoid of the target neoantigen (136) by means of chromosomal deletion. In another study, expression of the target was not only reduced by loss of the mutant alleles but also by a global downregulation of target gene expression (137). Some tumor entities including checkpoint-inhibition refractory pancreatic duct adenocarcinoma may escape neoantigen-targeted vaccination therapy simply by inducing a potent local immunosuppressive milieu (138) that prevents activation of neoantigen-specific T cells. The most frequent escape mechanism, however, has been shown to be the loss of global MHC expression, both after adoptive transfer of T cells and after neoantigen-directed vaccination (139). Loss of MHC class I expression may represent the most challenging escape mechanism resulting in complete abrogation of tumor recognition by cytotoxic T cells.

The evolution of escape mechanisms calls for the careful design of neoantigen-directed immunotherapies to avoid the selection of resistant subclones and to ensure successful vaccination. As an example, tumors characterized by a strong immunosuppressive micromilieu may be treated by combining neoantigen-targeting vaccines with chemotherapeutic regimens that deplete immunosuppressive Tregs or MDSC (e.g., cyclophosphamide and gemcitabine, respectively). To enhance the efficacy of the vaccine and to break local immunotolerance, checkpoint inhibitors have already been used either in combination or after neoantigen vaccination, resulting in complete tumor regression in a number of treated patients (50). The combination of neoantigen vaccines and checkpoint inhibitors seems in many ways ideal since these novel vaccines induce high numbers of tumor-specific T cells whose cytotoxic function can be restored by coadministration of checkpoint inhibitors. To prevent the selection of tumor clones with downregulated target antigen the choice of the neoantigen targets seem critical. In contrast to monoclonal immune responses, polyclonal immune responses against multiple neoantigens have been shown in murine tumor models to reduce the formation of escape variants (140). Ideally, the spectrum of neoantigens is to include driver mutations in genes with essential functions in tumor cell vitality, proliferation or metastasis. However, since driver mutations are not only rare but also rarely immunogenic in the context of a given MHC haplotype these mutations have so far not been used frequently in the context of neoantigen vaccines.

The emergence of tumor subclones devoid of MHC class I expression represents the most challenging resistance mechanism to vaccines so far. Allelic loss of MHC molecules prevents recognition of the tumor cells by CD8 T cells and, in some cases, even CD4 T cells. In contrast, downregulation of MHC molecules can be counteracted by small molecules such as cobimetinib which is currently under clinical investigation in combination with the PD-L1 targeting antibody atezolizumab for the treatment of colorectal cancer (trial number: NCT01988896). A critical question to be considered in the design of vaccines is whether MHC-negative tumor clones are already present at the beginning of the vaccination or if the resistant clones emerge during vaccination. If resistant clones emerge during vaccination, then vaccination should be designed to inflict maximum damage in a short period of time to avoid the equilibrium and the escape phase of the tumor-immune cell interaction. According to this hypothesis, an ideal vaccination regimen would consist of a limited number of vaccinations with a maximized magnitude of the ensuing T cell response (“hit hard and early”). Even in the case that MHC-negative tumor subclones are already present at the beginning of the vaccination, a “hit hard and early” vaccination might have advantages since high magnitude immune responses may favor the influx of natural killer cells which preferentially recognize and eliminate MHC-negative tumor cells. MHC-negative tumor cells should be preferred targets of natural killer cells. Consequently, it should be considered to combine neoantigen-directed immunotherapy with systemic NK cell activators. In preclinical studies, antibodies targeting NK cell checkpoints, such as CD96 have demonstrated the ability to control metastasis (141). In addition to the engagement of natural killer cells, CAR T cells could be used to overcome MHC-restriction and restore sensitivity to immunotherapy. A more simple approach to newly emerging MHC-negative tumor subclones or metastases may be to perform surgical resection whenever possible. Since the risk for the formation of MHC-negative tumor cells may correlate positively with tumor mass. This is consistent with results of mathematic models on targeted therapies which suggest that the likelihood of resistance following targeted therapy is a straight correlate of the number of tumor cells present at therapy start (142). Therefore, the combination of neoantigen vaccines with surgery or alternative cytoreductive means seems to be critical to minimize the risk of resistant tumor cells. The use of vaccines as an adjuvant treatment following surgery seems ideal since removal of the tumor abrogates the tumor-mediated immunosuppression and minimizes the number of post-operative tumor cells and therefore the chance for the survival of MHC-negative clones. In contrast to current chemotherapeutic regimens the combined treatment of surgery and tumor vaccinations may be a valuable option even in patients with advanced/metastatic disease since resistance to vaccinations is frequently a local instead of a systemic challenge. Finally, strategies targeting neoantigens could be of therapeutic value even in neoadjuvant settings. Consistent with this assumption, it has been shown in murine models with resectable tumors that neoadjuvant T cell stimulation using antibodies targeting PD-1 and CD137 was more effective in preventing metastasis compared with the same treatment when applied after tumor resection (143). These results suggest that under certain circumstances the tumor may serve as an important source of immunogenic antigens that can be exploited to induce neoantigen-specific immune responses.

In summary, the advent of novel therapies targeting neoantigens will revolutionize the treatment of cancer patients in the decades to come by fulfilling the promise of a personalized, individual treatment. Vaccinations are ideally suited for combination therapies, particularly in combination with checkpoint inhibitors, but also in combination with surgery, radiation therapy, chemotherapy, and locoregional and locally ablative procedures.

## Author Contributions

TW and FK planned the manuscript and wrote sections of the manuscript. Both authors read and approved the submitted version of the manuscript.

## Conflict of Interest Statement

The authors declare that the research was conducted in the absence of any commercial or financial relationships that could be construed as a potential conflict of interest.

## References

[1] Druker BJ Talpaz M Resta DJ Peng B Buchdunger E Ford JM Efficacy and safety of a specific inhibitor of the BCR-ABL tyrosine kinase in chronic myeloid leukemia. N Engl J Med (2001) 344:1031–7. 10.1056/NEJM200104053441401 11287972

[2] Coley WB The treatment of malignant tumors by repeated inoculations of erysipelas. With a report of ten original cases. 1893. Clin Orthop Relat Res (1991) 262:3–11. 1984929

[3] Starnes CO Coley’s toxins in perspective. Nature (1992) 357:11–2. 10.1038/357011a0 1574121

[4] Waldmann TA Immunotherapy: past, present and future. Nat Med (2003) 9:269–77. 10.1038/nm0303-269 12612576

[5] Foley EJ Antigenic properties of methylcholanthrene-induced tumors in mice of the strain of origin. Cancer Res (1953) 13:835–7. 13116120

[6] Prehn RT Main JM Immunity to methylcholanthrene-induced sarcomas. J Natl Cancer Inst (1957) 18:769–78. 13502695

[7] Klein G Sjogren HO Klein E Hellstrom KE Demonstration of resistance against methylcholanthrene-induced sarcomas in the primary autochthonous host. Cancer Res (1960) 20:1561–72. 13756652

[8] Steinman RM Witmer MD. Lymphoid dendritic cells are potent stimulators of the primary mixed leukocyte reaction in mice. Proc Natl Acad Sci U S A (1978) 75:5132–6. 10.1073/pnas.75.10.5132 154105 PMC336278

[9] Babbitt BP Allen PM Matsueda G Haber E Unanue ER. Binding of immunogenic peptides to Ia histocompatibility molecules. Nature (1985) 317:359–61. 10.1038/317359a0 3876513

[10] Lee JS Trowsdale J Bodmer WF. cDNA clones coding for the heavy chain of human HLA-DR antigen. Proc Natl Acad Sci U S A (1982) 79:545–9. 10.1073/pnas.79.2.545 6952207 PMC345781

[11] Gillis S Smith KA Long term culture of tumour-specific cytotoxic T cells. Nature (1977) 268:154–6. 10.1038/268154a0 145543

[12] Knuth A Danowski B Oettgen HF Old LJ. T-cell-mediated cytotoxicity against autologous malignant melanoma: analysis with interleukin 2-dependent T-cell cultures. Proc Natl Acad Sci U S A (1984) 81:3511–5. 10.1073/pnas.81.11.3511 6610177 PMC345538

[13] Svennevig JL Lunde OC Holter J Bjorgsvik D Lymphoid infiltration and prognosis in colorectal carcinoma. Br J Cancer (1984) 49:375–7. 10.1038/bjc.1984.60 6704315 PMC1976736

[14] Wada Y Nakashima O Kutami R Yamamoto O Kojiro M. Clinicopathological study on hepatocellular carcinoma with lymphocytic infiltration. Hepatology (1998) 27:407–14. 10.1002/hep.510270214 9462638

[15] Schwartzentruber DJ Hom SS Dadmarz R White DE Yannelli JR Steinberg SM In vitro predictors of therapeutic response in melanoma patients receiving tumor-infiltrating lymphocytes and interleukin-2. J Clin Oncol (1994) 12:1475–83. 10.1200/JCO.1994.12.7.1475 8021739

[16] Kang TW Yevsa T Woller N Hoenicke L Wuestefeld T Dauch D Senescence surveillance of pre-malignant hepatocytes limits liver cancer development. Nature (2011) 479:547–51. 10.1038/nature10599 22080947

[17] Shankaran V Ikeda H Bruce AT White JM Swanson PE Old LJ IFNgamma and lymphocytes prevent primary tumour development and shape tumour immunogenicity. Nature (2001) 410:1107–11. 10.1038/35074122 11323675

[18] Matsushita H Vesely MD Koboldt DC Rickert CG Uppaluri R Magrini VJ Cancer exome analysis reveals a T-cell-dependent mechanism of cancer immunoediting. Nature (2012) 482:400–4. 10.1038/nature10755 22318521 PMC3874809

[19] DuPage M Mazumdar C Schmidt LM Cheung AF Jacks T. Expression of tumour-specific antigens underlies cancer immunoediting. Nature (2012) 482:405–9. 10.1038/nature10803 22318517 PMC3288744

[20] Hanahan D Weinberg RA Hallmarks of cancer: the next generation. Cell (2011) 144:646–74. 10.1016/j.cell.2011.02.013 21376230

[21] Kantoff PW Higano CS Shore ND Berger ER Small EJ Penson DF Sipuleucel-T immunotherapy for castration-resistant prostate cancer. N Engl J Med (2010) 363:411–22. 10.1056/NEJMoa1001294 20818862

[22] Hodi FS O’Day SJ McDermott DF Weber RW Sosman JA Haanen JB Improved survival with ipilimumab in patients with metastatic melanoma. N Engl J Med (2010) 363:711–23. 10.1056/NEJMoa1003466 20525992 PMC3549297

[23] Wolchok JD Kluger H Callahan MK Postow MA Rizvi NA Lesokhin AM Nivolumab plus ipilimumab in advanced melanoma. N Engl J Med (2013) 369:122–33. 10.1056/NEJMoa1302369 23724867 PMC5698004

[24] Callahan MK Kluger H Postow MA Segal NH Lesokhin A Atkins MB Nivolumab plus ipilimumab in patients with advanced melanoma: updated survival, response, and safety data in a phase I dose-escalation study. J Clin Oncol (2017). 10.1200/JCO.2017.72.2850 29040030 PMC5946731

[25] Larkin J Chiarion-Sileni V Gonzalez R Grob JJ Cowey CL Lao CD Combined nivolumab and ipilimumab or monotherapy in untreated melanoma. N Engl J Med (2015) 373:23–34. 10.1056/NEJMoa1504030 26027431 PMC5698905

[26] Ansell SM Lesokhin AM Borrello I Halwani A Scott EC Gutierrez M PD-1 blockade with nivolumab in relapsed or refractory Hodgkin’s lymphoma. N Engl J Med (2015) 372:311–9. 10.1056/NEJMoa1411087 25482239 PMC4348009

[27] Barnd DL Lan MS Metzgar RS Finn OJ. Specific, major histocompatibility complex-unrestricted recognition of tumor-associated mucins by human cytotoxic T cells. Proc Natl Acad Sci U S A (1989) 86:7159–63. 10.1073/pnas.86.18.7159 2674949 PMC298015

[28] van der Bruggen P Traversari C Chomez P Lurquin C De PE Van den Eynde B A gene encoding an antigen recognized by cytolytic T lymphocytes on a human melanoma. Science (1991) 254:1643–7. 10.1126/science.1840703 1840703

[29] Kawashima I Tsai V Southwood S Takesako K Sette A Celis E. Identification of HLA-A3-restricted cytotoxic T lymphocyte epitopes from carcinoembryonic antigen and HER-2/neu by primary in vitro immunization with peptide-pulsed dendritic cells. Cancer Res (1999) 59:431–5. 9927058

[30] Parkhurst MR Yang JC Langan RC Dudley ME Nathan DA Feldman SA T cells targeting carcinoembryonic antigen can mediate regression of metastatic colorectal cancer but induce severe transient colitis. Mol Ther (2011) 19:620–6. 10.1038/mt.2010.272 21157437 PMC3048186

[31] Liang J Ding T Guo ZW Yu XJ Hu YZ Zheng L Expression pattern of tumour-associated antigens in hepatocellular carcinoma: association with immune infiltration and disease progression. Br J Cancer (2013) 109:1031–9. 10.1038/bjc.2013.390 23868000 PMC3749565

[32] Flecken T Schmidt N Hild S Gostick E Drognitz O Zeiser R Immunodominance and functional alterations of tumor-associated antigen-specific CD8+ T-cell responses in hepatocellular carcinoma. Hepatology (2014) 59:1415–26. 10.1002/hep.26731 24002931 PMC4139003

[33] Robbins PF Kassim SH Tran TL Crystal JS Morgan RA Feldman SA A pilot trial using lymphocytes genetically engineered with an NY-ESO-1-reactive T-cell receptor: long-term follow-up and correlates with response. Clin Cancer Res (2015) 21:1019–27. 10.1158/1078-0432.CCR-14-2708 25538264 PMC4361810

[34] Vogelstein B Papadopoulos N Velculescu VE Zhou S Diaz LA Jr Kinzler KW. Cancer genome landscapes. Science (2013) 339:1546–58. 10.1126/science.1235122 23539594 PMC3749880

[35] Greenman C Stephens P Smith R Dalgliesh GL Hunter C Bignell G Patterns of somatic mutation in human cancer genomes. Nature (2007) 446:153–8. 10.1038/nature05610 17344846 PMC2712719

[36] Bozic I Antal T Ohtsuki H Carter H Kim D Chen S Accumulation of driver and passenger mutations during tumor progression. Proc Natl Acad Sci U S A (2010) 107:18545–50. 10.1073/pnas.1010978107 20876136 PMC2972991

[37] Pinto S Sommermeyer D Michel C Wilde S Schendel D Uckert W Misinitiation of intrathymic MART-1 transcription and biased TCR usage explain the high frequency of MART-1-specific T cells. Eur J Immunol (2014) 44:2811–21. 10.1002/eji.201444499 24846220

[38] Wolfel T Hauer M Schneider J Serrano M Wolfel C Klehmann-Hieb E A p16INK4a-insensitive CDK4 mutant targeted by cytolytic T lymphocytes in a human melanoma. Science (1995) 269:1281–4. 10.1126/science.7652577 7652577

[39] Lennerz V Fatho M Gentilini C Frye RA Lifke A Ferel D The response of autologous T cells to a human melanoma is dominated by mutated neoantigens. Proc Natl Acad Sci U S A (2005) 102:16013–8. 10.1073/pnas.0500090102 16247014 PMC1266037

[40] Zhou J Dudley ME Rosenberg SA Robbins PF. Persistence of multiple tumor-specific T-cell clones is associated with complete tumor regression in a melanoma patient receiving adoptive cell transfer therapy. J Immunother (2005) 28:53–62. 10.1097/00002371-200501000-00007 15614045 PMC2175172

[41] Lynch HT de la Chapelle A Hereditary colorectal cancer. N Engl J Med (2003) 348:919–32. 10.1056/NEJMra012242 12621137

[42] Castle JC Kreiter S Diekmann J Lower M van de Roemer N de GJ Exploiting the mutanome for tumor vaccination. Cancer Res (2012) 72:1081–91. 10.1158/0008-5472.CAN-11-3722 22237626

[43] Karasaki T Nagayama K Kuwano H Nitadori JI Sato M Anraku M Prediction and prioritization of neoantigens: integration of RNA sequencing data with whole-exome sequencing. Cancer Sci (2017) 108:170–7. 10.1111/cas.13131 27960040 PMC5329159

[44] Alexandrov LB Nik-Zainal S Wedge DC Aparicio SA Behjati S Biankin AV Signatures of mutational processes in human cancer. Nature (2013) 500:415–21. 10.1038/nature12477 23945592 PMC3776390

[45] Fortier MH Caron E Hardy MP Voisin G Lemieux S Perreault C The MHC class I peptide repertoire is molded by the transcriptome. J Exp Med (2008) 205:595–610. 10.1084/jem.20071985 18299400 PMC2275383

[46] Engels B Engelhard VH Sidney J Sette A Binder DC Liu RB Relapse or eradication of cancer is predicted by peptide-major histocompatibility complex affinity. Cancer Cell (2013) 23:516–26. 10.1016/j.ccr.2013.03.018 23597565 PMC3658176

[47] Kammertoens T Blankenstein T. It’s the peptide-MHC affinity, stupid. Cancer Cell (2013) 23:429–31. 10.1016/j.ccr.2013.04.004 23597560

[48] Bassani-Sternberg M Braunlein E Klar R Engleitner T Sinitcyn P Audehm S Direct identification of clinically relevant neoepitopes presented on native human melanoma tissue by mass spectrometry. Nat Commun (2016) 7:13404. 10.1038/ncomms13404 27869121 PMC5121339

[49] Abelin JG Keskin DB Sarkizova S Hartigan CR Zhang W Sidney J Mass spectrometry profiling of HLA-associated peptidomes in mono-allelic cells enables more accurate epitope prediction. Immunity (2017) 46:315–26. 10.1016/j.immuni.2017.02.007 28228285 PMC5405381

[50] Sahin U Derhovanessian E Miller M Kloke BP Simon P Lower M Personalized RNA mutanome vaccines mobilize poly-specific therapeutic immunity against cancer. Nature (2017) 547:222–6. 10.1038/nature23003 28678784

[51] Ott PA Hu Z Keskin DB Shukla SA Sun J Bozym DJ An immunogenic personal neoantigen vaccine for patients with melanoma. Nature (2017) 547:217–21. 10.1038/nature22991 28678778 PMC5577644

[52] Carreno BM Magrini V Becker-Hapak M Kaabinejadian S Hundal J Petti AA Cancer immunotherapy. A dendritic cell vaccine increases the breadth and diversity of melanoma neoantigen-specific T cells. Science (2015) 348:803–8. 10.1126/science.aaa3828 25837513 PMC4549796

[53] Schmidt J Guillaume P Dojcinovic D Karbach J Coukos G Luescher I. In silico and cell-based analyses reveal strong divergence between prediction and observation of T-cell-recognized tumor antigen T-cell epitopes. J Biol Chem (2017) 292:11840–9. 10.1074/jbc.M117.789511 28536262 PMC5512077

[54] Albert ML Sauter B Bhardwaj N. Dendritic cells acquire antigen from apoptotic cells and induce class I-restricted CTLs. Nature (1998) 392:86–9. 10.1038/32183 9510252

[55] Broz ML Binnewies M Boldajipour B Nelson AE Pollack JL Erle DJ Dissecting the tumor myeloid compartment reveals rare activating antigen-presenting cells critical for T cell immunity. Cancer Cell (2014) 26:938 10.1016/j.ccell.2014.09.007 28898680

[56] Gros A Parkhurst MR Tran E Pasetto A Robbins PF Ilyas S Prospective identification of neoantigen-specific lymphocytes in the peripheral blood of melanoma patients. Nat Med (2016) 22:433–8. 10.1038/nm.4051 26901407 PMC7446107

[57] Cohen CJ Gartner JJ Horovitz-Fried M Shamalov K Trebska-McGowan K Bliskovsky VV Isolation of neoantigen-specific T cells from tumor and peripheral lymphocytes. J Clin Invest (2015) 125:3981–91. 10.1172/JCI82416 26389673 PMC4607110

[58] Bentzen AK Marquard AM Lyngaa R Saini SK Ramskov S Donia M Large-scale detection of antigen-specific T cells using peptide-MHC-I multimers labeled with DNA barcodes. Nat Biotechnol (2016) 34:1037–45. 10.1038/nbt.3662 27571370

[59] Wirth TC Xue HH Rai D Sabel JT Bair T Harty JT Repetitive antigen stimulation induces stepwise transcriptome diversification but preserves a core signature of memory CD8(+) T cell differentiation. Immunity (2010) 33:128–40. 10.1016/j.immuni.2010.06.014 20619696 PMC2912220

[60] Smith-Garvin JE Koretzky GA Jordan MS. T cell activation. Annu Rev Immunol (2009) 27:591–619. 10.1146/annurev.immunol.021908.132706 19132916 PMC2740335

[61] Slaney CY Kershaw MH Darcy PK. Trafficking of T cells into tumors. Cancer Res (2014) 74:7168–74. 10.1158/0008-5472.CAN-14-2458 25477332

[62] Caramalho I Faroudi M Padovan E Muller S Valitutti S. Visualizing CTL/melanoma cell interactions: multiple hits must be delivered for tumour cell annihilation. J Cell Mol Med (2009) 13:3834–46. 10.1111/j.1582-4934.2008.00586.x 19017355 PMC4516531

[63] Wong SQ Behren A Mar VJ Woods K Li J Martin C Whole exome sequencing identifies a recurrent RQCD1 P131L mutation in cutaneous melanoma. Oncotarget (2015) 6:1115–27. 10.18632/oncotarget.2747 25544760 PMC4359221

[64] Giannakis M Mu XJ Shukla SA Qian ZR Cohen O Nishihara R Genomic correlates of immune-cell infiltrates in colorectal carcinoma. Cell Rep (2016) 15(4):857–65. 10.1016/j.celrep.2016.10.009 PMC4850357 27149842

[65] Ionov Y Yamamoto H Krajewski S Reed JC Perucho M. Mutational inactivation of the proapoptotic gene BAX confers selective advantage during tumor clonal evolution. Proc Natl Acad Sci U S A (2000) 97:10872–7. 10.1073/pnas.190210897 10984511 PMC27116

[66] Inderberg EM Walchli S Myhre MR Trachsel S Almasbak H Kvalheim G T cell therapy targeting a public neoantigen in microsatellite instable colon cancer reduces in vivo tumor growth. Oncoimmunology (2017) 6:e1302631. 10.1080/2162402X.2017.1302631 28507809 PMC5414866

[67] Sveen A Johannessen B Tengs T Danielsen SA Eilertsen IA Lind GE Multilevel genomics of colorectal cancers with microsatellite instability-clinical impact of JAK1 mutations and consensus molecular subtype 1. Genome Med (2017) 9:46. 10.1186/s13073-017-0434-0 28539123 PMC5442873

[68] Bauer K Nelius N Reuschenbach M Koch M Weitz J Steinert G T cell responses against microsatellite instability-induced frameshift peptides and influence of regulatory T cells in colorectal cancer. Cancer Immunol Immunother (2013) 62:27–37. 10.1007/s00262-012-1303-8 22729559 PMC11029741

[69] Bouffet E Larouche V Campbell BB Merico D de BR Aronson M Immune checkpoint inhibition for hypermutant glioblastoma multiforme resulting from germline biallelic mismatch repair deficiency. J Clin Oncol (2016) 34:2206–11. 10.1200/JCO.2016.66.6552 27001570

[70] Johanns TM Miller CA Dorward IG Tsien C Chang E Perry A Immunogenomics of hypermutated glioblastoma: a patient with germline POLE deficiency treated with checkpoint blockade immunotherapy. Cancer Discov (2016) 6:1230–6. 10.1158/2159-8290.CD-16-0575 27683556 PMC5140283

[71] Eggink FA Van Gool IC Leary A Pollock PM Crosbie EJ Mileshkin L Immunological profiling of molecularly classified high-risk endometrial cancers identifies POLE-mutant and microsatellite unstable carcinomas as candidates for checkpoint inhibition. Oncoimmunology (2017) 6:e1264565 10.1080/2162402X.2016.1264565 28344870 PMC5353925

[72] Howitt BE Shukla SA Sholl LM Ritterhouse LL Watkins JC Rodig S Association of polymerase e-mutated and microsatellite-instable endometrial cancers with neoantigen load, number of tumor-infiltrating lymphocytes, and expression of PD-1 and PD-L1. JAMA Oncol (2015) 1:1319–23. 10.1001/jamaoncol.2015.2151 26181000

[73] Strickland KC Howitt BE Shukla SA Rodig S Ritterhouse LL Liu JF Association and prognostic significance of BRCA1/2-mutation status with neoantigen load, number of tumor-infiltrating lymphocytes and expression of PD-1/PD-L1 in high grade serous ovarian cancer. Oncotarget (2016) 7:13587–98. 10.18632/oncotarget.7277 26871470 PMC4924663

[74] Kloor M Michel S von Knebel DM. Immune evasion of microsatellite unstable colorectal cancers. Int J Cancer (2010) 127:1001–10. 10.1002/ijc.25283 20198617

[75] Nazemalhosseini ME Kashfi SM Mirtalebi H Taleghani MY Azimzadeh P Savabkar S Low level of microsatellite instability correlates with poor clinical prognosis in stage II colorectal cancer patients. J Oncol (2016) 2016:2196703. 10.1155/2016/2196703 27429617 PMC4939356

[76] Dienstmann R Mason MJ Sinicrope FA Phipps AI Tejpar S Nesbakken A Prediction of overall survival in stage II and III colon cancer beyond TNM system: a retrospective, pooled biomarker study. Ann Oncol (2017) 28:1023–31. 10.1093/annonc/mdx052 28453697 PMC5406760

[77] Maccaroni E Bracci R Giampieri R Bianchi F Belvederesi L Brugiati C Prognostic impact of mismatch repair genes germline defects in colorectal cancer patients: are all mutations equal? Oncotarget (2015) 6:38737–48. 10.18632/oncotarget.5395 26485756 PMC4770733

[78] Koi M Garcia M Choi C Kim HR Koike J Hemmi H Microsatellite alterations with allelic loss at 9p24.2 signify less-aggressive colorectal cancer metastasis. Gastroenterology (2016) 150:944–55. 10.1053/j.gastro.2015.12.032 26752111 PMC4808397

[79] Fujiyoshi K Yamamoto G Takenoya T Takahashi A Arai Y Yamada M Metastatic pattern of stage IV colorectal cancer with high-frequency microsatellite instability as a prognostic factor. Anticancer Res (2017) 37:239–47. 10.21873/anticanres.11313 28011498

[80] Tougeron D Mouillet G Trouilloud I Lecomte T Coriat R Aparicio T Efficacy of adjuvant chemotherapy in colon cancer with microsatellite instability: a large multicenter AGEO study. J Natl Cancer Inst (2016) 108:djv438 10.1093/jnci/djv438 26839356

[81] Tan WJ Hamzah JL Acharyya S Foo FJ Lim KH Tan IBH Evaluation of long-term outcomes of microsatellite instability status in an Asian Cohort of sporadic colorectal cancers. J Gastrointest Cancer (2017). 10.1007/s12029-017-9953-6 28550452

[82] Snyder A Makarov V Merghoub T Yuan J Zaretsky JM Desrichard A Genetic basis for clinical response to CTLA-4 blockade in melanoma. N Engl J Med (2014) 371:2189–99. 10.1056/NEJMoa1406498 25409260 PMC4315319

[83] Rizvi NA Hellmann MD Snyder A Kvistborg P Makarov V Havel JJ Cancer immunology. Mutational landscape determines sensitivity to PD-1 blockade in non-small cell lung cancer. Science (2015) 348:124–8. 10.1126/science.aaa1348 25765070 PMC4993154

[84] Van Allen EM Miao D Schilling B Shukla SA Blank C Zimmer L Genomic correlates of response to CTLA-4 blockade in metastatic melanoma. Science (2015) 350:207–11. 10.1126/science.aad0095 26359337 PMC5054517

[85] van Rooij N van Buuren MM Philips D Velds A Toebes M Heemskerk B Tumor exome analysis reveals neoantigen-specific T-cell reactivity in an ipilimumab-responsive melanoma. J Clin Oncol (2013) 31:e439–42. 10.1200/JCO.2012.47.7521 24043743 PMC3836220

[86] McGranahan N Furness AJ Rosenthal R Ramskov S Lyngaa R Saini SK Clonal neoantigens elicit T cell immunoreactivity and sensitivity to immune checkpoint blockade. Science (2016) 351:1463–9. 10.1126/science.aaf1490 26940869 PMC4984254

[87] Cho J Lee J Bang H Kim ST Park SH An JY Programmed cell death-ligand 1 expression predicts survival in patients with gastric carcinoma with microsatellite instability. Oncotarget (2017) 8:13320–8. 10.18632/oncotarget.14519 28076847 PMC5355099

[88] Dudley JC Lin MT Le DT Eshleman JR. Microsatellite instability as a biomarker for PD-1 blockade. Clin Cancer Res (2016) 22:813–20. 10.1158/1078-0432.CCR-15-1678 26880610

[89] Rosenbaum MW Bledsoe JR Morales-Oyarvide V Huynh TG Mino-Kenudson M. PD-L1 expression in colorectal cancer is associated with microsatellite instability, BRAF mutation, medullary morphology and cytotoxic tumor-infiltrating lymphocytes. Mod Pathol (2016) 29:1104–12. 10.1038/modpathol.2016.95 27198569

[90] Linnemann C van Buuren MM Bies L Verdegaal EM Schotte R Calis JJ High-throughput epitope discovery reveals frequent recognition of neo-antigens by CD4+ T cells in human melanoma. Nat Med (2015) 21:81–5. 10.1038/nm.3773 25531942

[91] Robbins PF Lu YC El-Gamil M Li YF Gross C Gartner J Mining exomic sequencing data to identify mutated antigens recognized by adoptively transferred tumor-reactive T cells. Nat Med (2013) 19:747–52. 10.1038/nm.3161 23644516 PMC3757932

[92] Shono Y Tanimura H Iwahashi M Tsunoda T Tani M Tanaka H Specific T-cell immunity against Ki-ras peptides in patients with pancreatic and colorectal cancers. Br J Cancer (2003) 88:530–6. 10.1038/sj.bjc.6600697 12592366 PMC2377177

[93] Ichiki Y Takenoyama M Mizukami M So T Sugaya M Yasuda M Simultaneous cellular and humoral immune response against mutated p53 in a patient with lung cancer. J Immunol (2004) 172:4844–50. 10.4049/jimmunol.172.8.4844 15067062

[94] Sharkey MS Lizee G Gonzales MI Patel S Topalian SL. CD4(+) T-cell recognition of mutated B-RAF in melanoma patients harboring the V599E mutation. Cancer Res (2004) 64:1595–9. 10.1158/0008-5472.CAN-03-3231 14996715

[95] Andersen MH Fensterle J Ugurel S Reker S Houben R Guldberg P Immunogenicity of constitutively active V599EBRaf. Cancer Res (2004) 64:5456–60. 10.1158/0008-5472.CAN-04-0937 15289355

[96] Holmstrom MO Hjortso MD Ahmad SM Met O Martinenaite E Riley C The JAK2V617F mutation is a target for specific T cells in the JAK2V617F-positive myeloproliferative neoplasms. Leukemia (2017) 31:495–8. 10.1038/leu.2016.290 27761006

[97] Holmstrom MO Martinenaite E Ahmad SM Met O Friese C Kjaer L The calreticulin (CALR) exon 9 mutations are promising targets for cancer immune therapy. Leukemia (2017). 10.1038/leu.2017.214 28676668

[98] Schwartzentruber J Korshunov A Liu XY Jones DT Pfaff E Jacob K Driver mutations in histone H3.3 and chromatin remodelling genes in paediatric glioblastoma. Nature (2012) 482:226–31. 10.1038/nature10833 22286061

[99] Bender S Tang Y Lindroth AM Hovestadt V Jones DT Kool M Reduced H3K27me3 and DNA hypomethylation are major drivers of gene expression in K27M mutant pediatric high-grade gliomas. Cancer Cell (2013) 24:660–72. 10.1016/j.ccr.2013.10.006 24183680

[100] Ochs K Ott M Bunse T Sahm F Bunse L Deumelandt K K27M-mutant histone-3 as a novel target for glioma immunotherapy. Oncoimmunology (2017) 6:e1328340. 10.1080/2162402X.2017.1328340 28811969 PMC5543817

[101] Schumacher T Bunse L Pusch S Sahm F Wiestler B Quandt J A vaccine targeting mutant IDH1 induces antitumour immunity. Nature (2014) 512:324–7. 10.1038/nature13387 25043048

[102] Hartmaier RJ Charo J Fabrizio D Goldberg ME Albacker LA Pao W Genomic analysis of 63,220 tumors reveals insights into tumor uniqueness and targeted cancer immunotherapy strategies. Genome Med (2017) 9:16. 10.1186/s13073-017-0408-2 28231819 PMC5324279

[103] Le DT Uram JN Wang H Bartlett BR Kemberling H Eyring AD PD-1 blockade in tumors with mismatch-repair deficiency. N Engl J Med (2015) 372:2509–20. 10.1056/NEJMoa1500596 26028255 PMC4481136

[104] Gubin MM Zhang X Schuster H Caron E Ward JP Noguchi T Checkpoint blockade cancer immunotherapy targets tumour-specific mutant antigens. Nature (2014) 515:577–81. 10.1038/nature13988 25428507 PMC4279952

[105] Yadav M Jhunjhunwala S Phung QT Lupardus P Tanguay J Bumbaca S Predicting immunogenic tumour mutations by combining mass spectrometry and exome sequencing. Nature (2014) 515:572–6. 10.1038/nature14001 25428506

[106] Woller N Gurlevik E Fleischmann-Mundt B Schumacher A Knocke S Kloos AM Viral infection of tumors overcomes resistance to PD-1-immunotherapy by broadening neoantigenome-directed T-cell responses. Mol Ther (2015) 23:1630–40. 10.1038/mt.2015.115 26112079 PMC4817928

[107] Duan F Duitama J Al SS Ayres CM Corcelli SA Pawashe AP Genomic and bioinformatic profiling of mutational neoepitopes reveals new rules to predict anticancer immunogenicity. J Exp Med (2014) 211:2231–48. 10.1084/jem.20141308 25245761 PMC4203949

[108] Gnjatic S Atanackovic D Jager E Matsuo M Selvakumar A Altorki NK Survey of naturally occurring CD4+ T cell responses against NY-ESO-1 in cancer patients: correlation with antibody responses. Proc Natl Acad Sci U S A (2003) 100:8862–7. 10.1073/pnas.1133324100 12853579 PMC166404

[109] Knocke S Fleischmann-Mundt B Saborowski M Manns MP Kuhnel F Wirth TC Tailored tumor immunogenicity reveals regulation of CD4 and CD8 T cell responses against cancer. Cell Rep (2016) 17:2234–46. 10.1016/j.celrep.2016.10.086 27880900

[110] Tran E Turcotte S Gros A Robbins PF Lu YC Dudley ME Cancer immunotherapy based on mutation-specific CD4+ T cells in a patient with epithelial cancer. Science (2014) 344:641–5. 10.1126/science.1251102 24812403 PMC6686185

[111] Kreiter S Vormehr M van de Roemer N Diken M Lower M Diekmann J Mutant MHC class II epitopes drive therapeutic immune responses to cancer. Nature (2015) 520:692–6. 10.1038/nature14426 25901682 PMC4838069

[112] Martin SD Brown SD Wick DA Nielsen JS Kroeger DR Twumasi-Boateng K Low mutation burden in ovarian cancer may limit the utility of neoantigen-targeted vaccines. PLoS One (2016) 11:e0155189. 10.1371/journal.pone.0155189 27192170 PMC4871527

[113] Diken M Vormehr M Grunwitz C Kreiter S Tureci O Sahin U. Discovery and subtyping of neo-epitope specific T-cell responses for cancer immunotherapy: addressing the mutanome. Methods Mol Biol (2017) 1499:223–36. 10.1007/978-1-4939-6481-9_14 27987153

[114] Rosenberg SA Yannelli JR Yang JC Topalian SL Schwartzentruber DJ Weber JS Treatment of patients with metastatic melanoma with autologous tumor-infiltrating lymphocytes and interleukin 2. J Natl Cancer Inst (1994) 86:1159–66. 10.1093/jnci/86.15.1159 8028037

[115] Johnson LA Morgan RA Dudley ME Cassard L Yang JC Hughes MS Gene therapy with human and mouse T-cell receptors mediates cancer regression and targets normal tissues expressing cognate antigen. Blood (2009) 114:535–46. 10.1182/blood-2009-03-211714 19451549 PMC2929689

[116] Leisegang M Engels B Schreiber K Yew PY Kiyotani K Idel C Eradication of large solid tumors by gene therapy with a T-cell receptor targeting a single cancer-specific point mutation. Clin Cancer Res (2016) 22:2734–43. 10.1158/1078-0432.CCR-15-2361 26667491 PMC4891260

[117] Leisegang M Kammertoens T Uckert W Blankenstein T. Targeting human melanoma neoantigens by T cell receptor gene therapy. J Clin Invest (2016) 126:854–8. 10.1172/JCI83465 26808500 PMC4767365

[118] Parkhurst M Gros A Pasetto A Prickett T Crystal JS Robbins P Isolation of T-cell receptors specifically reactive with mutated tumor-associated antigens from tumor-infiltrating lymphocytes based on CD137 expression. Clin Cancer Res (2017) 23:2491–505. 10.1158/1078-0432.CCR-16-2680 27827318 PMC6453117

[119] Deniger DC Pasetto A Tran E Parkhurst MR Cohen CJ Robbins PF Stable, nonviral expression of mutated tumor neoantigen-specific T-cell receptors using the sleeping beauty transposon/transposase system. Mol Ther (2016) 24:1078–89. 10.1038/mt.2016.51 26945006 PMC4923320

[120] Stronen E Toebes M Kelderman S van Buuren MM Yang W van RN Targeting of cancer neoantigens with donor-derived T cell receptor repertoires. Science (2016) 352:1337–41. 10.1126/science.aaf2288 27198675

[121] Posey AD Jr Schwab RD Boesteanu AC Steentoft C Mandel U Engels B Engineered CAR T cells targeting the cancer-associated Tn-glycoform of the membrane mucin MUC1 control adenocarcinoma. Immunity (2016) 44:1444–54. 10.1016/j.immuni.2016.05.014 27332733 PMC5358667

[122] Dunn GP Old LJ Schreiber RD. The three Es of cancer immunoediting. Annu Rev Immunol (2004) 22:329–60. 10.1146/annurev.immunol.22.012703.104803 15032581

[123] Riker A Cormier J Panelli M Kammula U Wang E Abati A Immune selection after antigen-specific immunotherapy of melanoma. Surgery (1999) 126:112–20. 10.1016/S0039-6060(99)70143-1 10455872

[124] Olson BM McNeel DG. Antigen loss and tumor-mediated immunosuppression facilitate tumor recurrence. Expert Rev Vaccines (2012) 11:1315–7. 10.1586/erv.12.107 23249231 PMC3557828

[125] Donia M Harbst K van BM Kvistborg P Lindberg MF Andersen R Acquired immune resistance follows complete tumor regression without loss of target antigens or IFNgamma signaling. Cancer Res (2017) 77:4562–6. 10.1158/0008-5472.CAN-16-3172 28655789

[126] Natali PG Giacomini P Bigotti A Imai K Nicotra MR Ng AK Heterogeneity in the expression of HLA and tumor-associated antigens by surgically removed and cultured breast carcinoma cells. Cancer Res (1983) 43:660–8. 6336658

[127] Garrido F Ruiz-Cabello F Aptsiauri N. Rejection versus escape: the tumor MHC dilemma. Cancer Immunol Immunother (2017) 66:259–71. 10.1007/s00262-016-1947-x 28040849 PMC11028748

[128] Seliger B Harders C Lohmann S Momburg F Urlinger S Tampe R Down-regulation of the MHC class I antigen-processing machinery after oncogenic transformation of murine fibroblasts. Eur J Immunol (1998) 28:122–33. 10.1002/(SICI)1521-4141(199801)28:01<122::AID-IMMU122>3.0.CO;2-F 9485192

[129] Restifo NP Marincola FM Kawakami Y Taubenberger J Yannelli JR Rosenberg SA. Loss of functional beta 2-microglobulin in metastatic melanomas from five patients receiving immunotherapy. J Natl Cancer Inst (1996) 88:100–8. 10.1093/jnci/88.2.100 8537970 PMC2248456

[130] Spiotto MT Rowley DA Schreiber H. Bystander elimination of antigen loss variants in established tumors. Nat Med (2004) 10:294–8. 10.1038/nm999 14981514

[131] Joyce JA Fearon DT. T cell exclusion, immune privilege, and the tumor microenvironment. Science (2015) 348:74–80. 10.1126/science.aaa6204 25838376

[132] Zou W. Immunosuppressive networks in the tumour environment and their therapeutic relevance. Nat Rev Cancer (2005) 5:263–74. 10.1038/nrc1586 15776005

[133] Seton-Rogers S Pancreatic cancer: dodging immunosuppression. Nat Rev Cancer (2016) 16:480–1. 10.1038/nrc.2016.80 27451955

[134] Turley SJ Cremasco V Astarita JL. Immunological hallmarks of stromal cells in the tumour microenvironment. Nat Rev Immunol (2015) 15:669–82. 10.1038/nri3902 26471778

[135] Wherry EJ Kurachi M. Molecular and cellular insights into T cell exhaustion. Nat Rev Immunol (2015) 15:486–99. 10.1038/nri3862 26205583 PMC4889009

[136] Anagnostou V Smith KN Forde PM Niknafs N Bhattacharya R White J Evolution of neoantigen landscape during immune checkpoint blockade in non-small cell lung cancer. Cancer Discov (2017) 7:264–76. 10.1158/2159-8290.CD-16-0828 28031159 PMC5733805

[137] Verdegaal EM de Miranda NF Visser M Harryvan T van Buuren MM Andersen RS Neoantigen landscape dynamics during human melanoma-T cell interactions. Nature (2016) 536:91–5. 10.1038/nature18945 27350335

[138] Bailey P Chang DK Forget MA Lucas FA Alvarez HA Haymaker C Exploiting the neoantigen landscape for immunotherapy of pancreatic ductal adenocarcinoma. Sci Rep (2016) 6:35848. 10.1038/srep35848 27762323 PMC5071896

[139] Schrors B Lubcke S Lennerz V Fatho M Bicker A Wolfel C HLA class I loss in metachronous metastases prevents continuous T cell recognition of mutated neoantigens in a human melanoma model. Oncotarget (2017) 8:28312–27. 10.18632/oncotarget.16048 28423700 PMC5438652

[140] Pulido J Kottke T Thompson J Galivo F Wongthida P Diaz RM Using virally expressed melanoma cDNA libraries to identify tumor-associated antigens that cure melanoma. Nat Biotechnol (2012) 30:337–43. 10.1038/nbt.2157 22426030 PMC3891505

[141] Blake SJ Stannard K Liu J Allen S Yong MC Mittal D Suppression of metastases using a new lymphocyte checkpoint target for cancer immunotherapy. Cancer Discov (2016) 6:446–59. 10.1158/2159-8290.CD-15-0944 26787820

[142] Bozic I Reiter JG Allen B Antal T Chatterjee K Shah P Evolutionary dynamics of cancer in response to targeted combination therapy. Elife (2013) 2:e00747. 10.7554/eLife.00747 23805382 PMC3691570

[143] Liu J Blake SJ Yong MC Harjunpaa H Ngiow SF Takeda K Improved efficacy of neoadjuvant compared to adjuvant immunotherapy to eradicate metastatic disease. Cancer Discov (2016) 6:1382–99. 10.1158/2159-8290.CD-16-0577 27663893

